# Bridging the Language Gap in Healthcare: A Narrative Review of Interpretation Services and Access to Care for Immigrants and Refugees in Greece and Europe

**DOI:** 10.3390/healthcare14020215

**Published:** 2026-01-15

**Authors:** Athina Pitta, Maria Tzitiridou-Chatzopoulou, Arsenios Tsiotsias, Serafeim Savvidis

**Affiliations:** Department of Midwifery, University of Western Macedonia, 50 200 Ptolemaida, Greece

**Keywords:** language barriers, medical interpretation, intercultural mediation, migrant and refugee health, healthcare access, Greece

## Abstract

**Highlights:**

**What are the main findings?**

- Professional interpreters and intercultural mediators substantially improve communication accuracy, healthcare access, patient satisfaction, and clinical outcomes for migrants and refugees, outperforming ad hoc interpretation by a wide margin.
- Limited proficiency in Greek is strongly associated with longer waiting times, reduced utilization of services, poorer comprehension of medical information, and diminished patient satisfaction, underscoring language as a critical determinant of healthcare inequity in Greece.

**What are the implications of the main findings?**

- Implementing structured, professionally staffed interpretation services is essential for ensuring equitable and safe healthcare delivery, reducing clinical errors, shortening hospital stays, and contributing to more efficient use of healthcare resources.
- Integrating intercultural mediation and systematic linguistic support into national healthcare policy can strengthen patient safety, enhance trust in health services, and support the effective integration of migrants and refugees into the Greek healthcare system.

**Abstract:**

**Background**: Language barriers remain a major obstacle to equitable healthcare access for immigrants and refugees across Europe. Greece, as both a transit and host country, faces persistent challenges in providing linguistically and culturally appropriate care. **Methods**: This study presents a narrative literature review synthesizing international, European, and Greek evidence on the effects of limited language proficiency, professional interpretation, and intercultural mediation on healthcare access, patient safety, satisfaction, and clinical outcomes. Peer-reviewed studies and selected grey literature were identified through searches of PubMed, Scopus, Web of Science, and CINAHL. **Results**: The evidence consistently demonstrates that the absence of professional interpretation is associated with substantially higher rates of clinically significant communication errors, longer hospital stays, increased readmissions, and higher healthcare costs. In contrast, the use of trained medical interpreters and intercultural mediators improves comprehension, shared decision-making, patient satisfaction, and clinical outcomes. Comparative European data from Italy, Spain, Germany, and Sweden show that institutionalized interpretation systems outperform Greece’s fragmented, NGO-dependent approach. Greek studies further reveal that limited proficiency in Greek is associated with reduced service utilization, longer waiting times, and lower patient satisfaction. **Conclusions**: This narrative review highlights the urgent need for Greece to adopt a coordinated, professionally staffed interpretation and intercultural mediation framework. Strengthening linguistic support within the healthcare system is essential for improving patient safety, equity, efficiency, and the integration of migrant and refugee populations.

## 1. Introduction

International migration has intensified over recent decades, reshaping the demographic and cultural landscape of healthcare systems across Europe. Greece has experienced sustained migration flows since the 1990s, further intensified by the refugee arrivals of 2015–2016, positioning the country as both a transit and host state for diverse migrant and refugee populations [1]. As a result, the Greek healthcare system faces ongoing challenges in ensuring equitable access to services that are linguistically and culturally appropriate.

In this review, the term migrants refers broadly to individuals who have relocated across borders for economic, educational, or family-related reasons, whereas refugees and asylum seekers denote populations displaced by conflict, persecution, or humanitarian crises, whose legal status, health needs, and access barriers may differ substantially.

Among the multiple barriers affecting migrant and refugee healthcare access, language discordance between patients and healthcare providers remains one of the most consequential. Limited proficiency in the host-country language compromises symptom reporting, diagnostic accuracy, shared decision-making, and adherence to treatment, thereby increasing the risk of clinical errors and adverse outcomes [2,3]. International evidence consistently shows that migrants and refugees with limited language proficiency receive lower-quality care than native-speaking populations, even when socioeconomic factors are taken into account [4,5].

Despite this well-established evidence, the organization of interpretation services within healthcare systems varies widely across Europe. Countries such as Italy, Spain, Germany, and Sweden have implemented institutionalized models of professional interpretation and intercultural mediation, supported by public funding or legal entitlements. These models have been associated with measurable improvements in patient satisfaction, communication accuracy, clinical outcomes, and healthcare efficiency [5,6,7,8]. In contrast, Greece continues to rely largely on fragmented, ad hoc, and NGO-dependent language support, resulting in inconsistent access to interpretation across regions and healthcare settings [9].

Although several Greek studies have documented the association between limited Greek proficiency and reduced healthcare utilization, longer waiting times, and lower patient satisfaction, the existing literature remains dispersed and insufficiently synthesized [10,11,12]. Moreover, Greek evidence is rarely situated within a broader European comparative framework, limiting its usefulness for policy development and system-level reform.

The purpose of this narrative review is therefore threefold:

(1) to synthesize international, European, and Greek evidence on the impact of language barriers on healthcare access, safety, and outcomes;
(2) to examine the effectiveness of professional interpretation and intercultural mediation services;
(3) to situate Greece within a comparative European context in order to identify policy gaps and transferable best practices.

By integrating quantitative clinical indicators—such as communication error rates, length of stay, and readmission patterns—with qualitative and policy-oriented evidence, this review aims to strengthen the empirical basis for institutionalizing linguistic and cultural support within the Greek healthcare system [2,13].

## 2. Conceptual Framework and Definitions

Language proficiency constitutes a decisive determinant of healthcare access and quality among migrant and refugee populations. Across diverse clinical environments, inadequate linguistic support is consistently associated with miscommunication, reduced adherence to treatment, diminished patient engagement, and heightened risk of clinical error [2,3]. To situate the analysis that follows, this section clarifies the core concepts underlying discussions of linguistic accessibility and culturally competent care.

Intercultural mediators are trained professionals who facilitate communication and mutual understanding between patients and health professionals across linguistic and cultural boundaries. Their contribution extends beyond the literal transfer of information to include the clarification of cultural norms, expectations, and explanatory models of illness; prevention and resolution of conflicts; support with navigating administrative and clinical systems; and the promotion of patient-centered care. Through these functions, mediators provide cultural clarification, psychosocial support, and relational continuity, thereby fostering conditions necessary for trustful and effective clinical encounters [14,15].

Professional medical interpreters, by contrast, are individuals formally employed to provide accurate, impartial, and confidential interpretation between a source and target language. These interpreters adhere to established standards of practice—including completeness, fidelity, role boundaries, and confidentiality—and their involvement has been repeatedly shown to reduce clinically significant errors, improve comprehension, and enhance satisfaction among both patients and clinicians [13,16]. In many settings, however, clinicians rely on ad hoc interpreters, such as family members, bilingual staff, or community volunteers. Although such arrangements may appear expedient, evidence demonstrates that they often result in omissions, distortions, and breaches of confidentiality and introduce substantial clinical and ethical risks [13,17].

These practices are commonly situated within the framework of culturally competent care (CCC), which emphasizes cultural awareness, communication skills, and organizational responsiveness to diversity [18]. However, recent scholarship has highlighted limitations of CCC when applied in isolation, particularly its tendency to focus on individual-level cultural traits rather than broader structural conditions. Complementary perspectives—such as cultural humility, which emphasizes reflexivity and power asymmetries; structural competence, which foregrounds legal, economic, and institutional determinants of health; and intersectional approaches that account for the interaction of language, gender, legal status, and socioeconomic position—offer additional analytical depth. Integrating these perspectives allows linguistic mediation to be understood not as a linear intervention but as one component within a complex system shaped by structural vulnerability, institutional trust, and health literacy.

More recent studies [19,20,21] conducted after 2018 further corroborate these associations while highlighting the growing role of digital and remote interpretation services. Evidence from European health systems indicates that tele-interpretation and digitally supported communication tools can mitigate language-related risks when in-person interpretation is unavailable, particularly during periods of system strain such as the COVID-19 pandemic. These studies [19,20,21] also emphasize the interaction between language barriers and health literacy, demonstrating that limited linguistic proficiency often co-occurs with reduced ability to navigate health information, consent procedures, and digital health platforms, thereby compounding clinical risk.

### 2.1. Language Barriers as a Determinant of Clinical Risk

A substantial body of international evidence shows that language discordance between clinicians and patients is not a minor inconvenience but a measurable determinant of compromised care quality, safety risk, and inefficient use of healthcare resources. Limited proficiency in the host-country language undermines the accuracy of clinical communication, weakens therapeutic relationships, and contributes to poorer health outcomes among migrant and refugee populations [3,4]. Quantitative studies highlight the scale of this problem. Flores et al. [17], in one of the most cited empirical examinations of interpretation quality, identified 396 interpretation errors across 57 pediatric encounters, 63% of which had potential clinical consequences. The frequency of such errors was dramatically higher when interpretation was provided by untrained individuals. By contrast, professional interpreters reduced clinically significant errors by an estimated 53–75% [13].

These communication failures extend far beyond misunderstandings of symptoms or diagnoses. Wilson et al. [3] reported that patients with limited language proficiency were significantly more likely to misinterpret medication instructions; more recent European studies similarly document persistent associations between language barriers, reduced comprehension, and adverse safety outcomes, including in digitally mediated care contexts [19,20,21]. The presence of trained interpreters has been shown to reduce inpatient length of stay by approximately 0.75 days [2] and to lower hospital readmission rates by 39% [13]. Such figures illustrate the direct clinical and operational consequences of inadequate linguistic support.

The financial costs of miscommunication are substantial. Duplicated diagnostic testing, avoidable complications, and prolonged hospitalizations generate an estimated USD 2000–6000 in additional healthcare expenditures per patient episode [13]. Ethical concerns also arise from reliance on ad hoc interpreters, whose involvement increases risks of distortion, omission, and confidentiality breaches [22]. Collectively, this evidence positions language discordance as a quantifiable—and modifiable—determinant of patient safety, clinical quality, and system-wide efficiency.

Although most quantitative estimates derive from non-Greek contexts, they remain highly relevant for Greece due to comparable patterns of linguistic exclusion; nevertheless, direct numerical transfer should be interpreted with caution.

### 2.2. Greece in European Comparative Context

Situating Greece within a broader European context reveals significant disparities in the institutionalization of medical interpretation and intercultural mediation. Although Greece faces demographic pressures comparable to those of other migration-affected states, its interpreter infrastructure remains comparatively fragmented and heavily reliant on NGOs.

Italy offers one of the most instructive comparative models. Several Italian regions—including Emilia–Romagna and Tuscany—have systematically integrated professional interpreters and intercultural mediators into maternity, primary, and hospital care. Evidence from these regions demonstrates that structured linguistic support reduces missed appointments by approximately 25% and increases patient satisfaction by 30–40% among migrant populations [7]. In contrast, interpreter availability in Greece varies widely between facilities and often depends on external organizations rather than coordinated public funding.

Spain also presents a relevant point of comparison. Regions such as Catalonia and the Basque Country have institutionalized interpreter provision in public healthcare, contributing to a 22–30% improvement in adherence to chronic disease management among migrant patients [6]. By contrast, Greek migrants with limited Greek proficiency report difficulty navigating basic health services and often avoid seeking care independently [10,12], underscoring unmet linguistic and structural needs.

Recent Southern European research has also drawn attention to the role of health literacy among migrant populations, particularly in contexts characterized by linguistic distance and complex administrative systems. Studies from Mediterranean countries highlight that limited health literacy, often intertwined with language barriers, constrains migrants’ ability to navigate healthcare pathways, understand preventive guidance, and engage with digital health services. These findings are especially relevant for Greece, where fragmented service provision and reliance on informal interpretation may amplify literacy-related inequities.

Germany’s model is characterized by widespread deployment of telephone and video interpretation services, despite inconsistent funding streams. Empirical studies from German hospitals indicate that LEP patients without access to interpretation face 70% higher odds of adverse clinical events and are twice as likely to misunderstand discharge instructions [5]. Remote interpretation in Germany has also been associated with reductions in length of stay comparable to those reported in other European and U.S. settings [2]. Greece, however, lacks a coordinated tele-interpretation infrastructure.

In northern Europe, Sweden demonstrates one of the most institutionalized models of linguistic support. Swedish law guarantees all patients access to free professional interpreters, and integration with electronic booking and medical record systems ensures consistent uptake. Research indicates that 96% of migrant women in maternity care report using interpreters during clinical encounters [8]. Such high levels of institutionalization stand in sharp contrast to the informal, inconsistent, and often improvised arrangements observed in Greece.

Taken together, these comparisons highlight the universality of language barriers across Europe while also revealing substantial differences in institutional responses. Countries with structured interpreter systems—including Italy, Spain, Germany, and Sweden—demonstrate measurable improvements in communication accuracy, satisfaction, adherence, and efficiency. For Greece, where limited Greek proficiency is strongly associated with restricted access, longer waiting times, and reduced satisfaction [10], the European evidence provides a compelling justification for transitioning from ad hoc, NGO-dependent services toward a professionalized and coordinated national model.

Key transferable lessons for Greece emerge from the comparative European evidence. These include the institutionalization of professional interpreter provision through stable public funding mechanisms, which ensures consistency and equity of access across healthcare settings. The integration of tele-interpretation systems represents a particularly effective strategy for improving linguistic support in rural, remote, and island regions where in-person interpreters are scarce. Establishing clear legal entitlements to professional interpretation can further standardize access, reduce reliance on ad hoc solutions, and strengthen patient safety. Finally, embedding trained intercultural mediators within high-impact clinical areas—such as maternity services, mental health care, and emergency departments—can address both linguistic and cultural barriers, enhancing communication quality, trust, and clinical effectiveness.

### 2.3. Greek Policy and Access to Care

Within both European and Greek legal frameworks, refugees and beneficiaries of international protection formally hold the right to healthcare access. However, practical access is shaped by resource availability, cultural and linguistic acceptability, navigation challenges, and pervasive communication barriers (World Health Organization [WHO] [23]). Greek studies show that strong command of Greek correlates with better knowledge and utilization of health services, whereas limited proficiency is associated with longer waiting times, reduced comprehension, and lower satisfaction [10]. These disparities are particularly pronounced in mental health services, where linguistic nuance and cultural framing are essential to diagnosis, therapeutic rapport, and treatment adherence [13].

These structural gaps directly inform the policy recommendations presented later in this review, underscoring the need for coordinated national action rather than fragmented or NGO-dependent solutions.

## 3. Materials and Methods

### 3.1. Review Design

This study adopts a narrative literature review design to synthesize evidence on the role of language proficiency, professional interpretation, and intercultural mediation in shaping healthcare access, quality, and outcomes for migrant and refugee populations. A narrative approach was selected due to the heterogeneity of study designs in this field, which includes quantitative clinical studies, qualitative research, mixed-methods analyses, and policy reports across multiple national contexts [13,15].

### 3.2. Search Strategy

Literature searches were conducted between January and March 2024 using PubMed, Scopus, Web of Science, and CINAHL. Grey literature from international organizations—including the World Health Organization [23] and the International Organization for Migration [24]—was also reviewed to contextualize policy and system-level evidence [23,24]. Reference lists of key studies were screened to identify additional relevant publications.

Search terms combined concepts related to language barriers and interpretation with healthcare and migration, including “language barriers,” “limited language proficiency,” “medical interpretation,” “intercultural mediation,” “migrant health,” “refugee health,” “Greece,” and “Europe.” Boolean operators and truncations were applied to refine the search.

### 3.3. Inclusion and Exclusion Criteria

Studies were included if they examined healthcare settings involving migrants, refugees, or asylum seekers and addressed language proficiency, interpretation services, or intercultural mediation in relation to healthcare access, communication quality, patient safety, or clinical outcomes [5,17]. Both empirical studies and systematic or narrative reviews were eligible.

Studies focusing exclusively on linguistic theory without healthcare application, non-migrant populations, or non-clinical contexts were excluded.

Grey literature was included only when produced by recognized institutions and when methodological transparency was sufficient to support analytical relevance.

### 3.4. Data Extraction and Synthesis

Titles and abstracts were screened for relevance, followed by full-text review of eligible studies. Data extraction focused on study design, country, population characteristics, type of language support (professional interpreters, ad hoc interpreters, intercultural mediators, tele-interpretation), and reported outcomes. Quantitative indicators—such as error rates, length of stay, readmissions, and costs—were extracted where available [2,13]. Qualitative findings on patient experience, trust, and cultural mediation were also incorporated [8,15].

Given the diversity of study designs and outcome measures, a formal meta-analysis was not feasible. Instead, findings were synthesized thematically to highlight patterns of convergence and divergence across national contexts and healthcare settings. Where findings across studies were conflicting, greater interpretive weight was given to studies with stronger methodological designs, larger samples, or clearer outcome measures.

## 4. Results

The results of this narrative review describe documented associations between language proficiency, interpreter use, and healthcare-related outcomes among migrant and refugee populations, as reported in international, European, and Greek studies. Across the reviewed literature, limited proficiency in the host-country language is examined in relation to communication processes, service utilization, and clinical indicators. The reported outcomes associated with different forms of language support are summarized in Table 1, Table 2, Table 3 and Table 4.

Professional interpreters consistently deliver high communication accuracy, reducing clinically significant errors and improving satisfaction for both patients and providers [13,17]. Their involvement is associated with shorter hospital stays, reductions in readmissions, and measurable cost savings [2,13]. Intercultural mediators extend these benefits by also addressing cultural norms and expectations, thereby enhancing trust and improving outcomes in sensitive clinical domains such as mental health and perinatal care [15,18]. Conversely, ad hoc interpreters—family members, bilingual staff, or untrained community members—are associated with frequent omissions, inaccuracies, confidentiality breaches, and a higher risk of adverse outcomes [17,22].

Collectively, Table 1 illustrates that structured, professionalized language support is superior to informal alternatives across all domains of care.

### 4.1. Quantitative Effects of Interpretation Services on Clinical Outcomes

Quantitative studies included in the review report associations between interpretation services and communication accuracy, patient comprehension, and selected utilization indicators, although effect sizes vary by population group, care setting, and study design. Reported reductions in clinically significant interpretation errors (approximately 53–75%) are primarily derived from hospital-based studies involving pediatric or general medicine populations, while estimates of reduced length of stay and readmission rates are contingent on institutional context and the availability of integrated interpreter services. These methodological differences should be considered when interpreting aggregated quantitative estimates.

Hospital-based studies describe associations between interpreter use and utilization indicators. These include reports of shorter inpatient length of stay among patients who received professional interpretation [2] and lower 30-day readmission rates in settings where enhanced interpreter services were available [13]. Financial analyses included in the reviewed literature describe differences in healthcare costs related to duplicated diagnostic testing and extended hospitalizations in the absence of adequate linguistic support [13]. Quantitative findings across outcome domains are summarized in Table 2.

**Table 2:** Quantitative Impact of Interpretation Services on Clinical Outcomes.

Outcome Domain	Professional Interpreters	Ad Hoc/No Interpreter	Key Sources
Communication Accuracy	53–75% reduction in clinically significant errors	5–8× higher error rates	[13,17]
Patient Comprehension	Clear understanding of diagnosis and treatment	LEP patients 4.5× more likely to misunderstand medication instructions	[3]
Shared Decision-Making	Higher engagement and autonomy	Reduced participation in decisions	[4]
Length of Stay (LOS)	Reduced by ~0.75 days	Prolonged LOS due to communication delays	[2]
Readmission Rates	39% reduction in 30-day readmissions	Higher readmission risk	[13]
Healthcare Costs	USD 2000–6000 savings per patient	Increased costs due to duplicated tests/extended stays	[5]
Safety Events	Fewer adverse events	70% higher odds of adverse events	[28]
Maternity/Perinatal Outcomes	Higher satisfaction, fewer crises	35–55% more communication problems	[7,8]

### 4.2. Comparative Findings Across Non-English-Speaking European Countries

Comparative studies and policy reports describe variation in the organization and availability of interpretation services across European healthcare systems. In Italy, regional programs incorporating professional interpreters and intercultural mediators are reported in association with changes in appointment attendance and patient-reported satisfaction [7]. Studies from Spain describe regional interpreter programs linked to reported differences in adherence to chronic disease management among migrant patients [6].

German studies document the use of telephone and video interpretation in hospital settings and report associations with communication-related adverse events and discharge comprehension [5]. In Sweden, legal provisions guaranteeing access to professional interpreters are reflected in high reported rates of interpreter use in maternity care settings [8]. In contrast, Greek studies describe variability in interpreter availability and reliance on informal arrangements, with reported associations involving service utilization, waiting times, and patient satisfaction among migrants with limited Greek proficiency [10,12]. These cross-national findings are summarized in Table 3.

**Table 3:** Comparison of National Interpreter and Mediation Policies (Greece and EU Countries).

Country	Policy Framework	Access to Interpreters	Funding Model	Documented Outcomes	Relevance to Greece
Greece	No national mandate; fragmented NGO-dependent	Inconsistent; often informal	External/NGO-based	Lower service use; poor satisfaction	Highlights need for national policy
Italy	Regional intercultural mediation laws	High availability	Regional public funding	25% missed appointments; satisfaction	Model for decentralized reform
Spain	Regional interpreter programs	Strong in Catalonia/Basque Country	Regional public funding	22–30% care adherence	Demonstrates benefit of structured access
Germany	Tele-interpretation widespread	High technical access	Mixed funding	70% adverse events	Model for tele-interpretation infrastructure
Sweden	Legal right to free interpreters	Near-universal uptake	State-funded	96% interpreter use in maternity care	Benchmark for institutionalization

### 4.3. Variation in Healthcare Experiences Among Migrant and Refugee Groups

Studies included in the review also report variation in communication needs and healthcare experiences across different migrant and refugee populations. Research focusing on Afghan and Syrian refugees describes language barriers in combination with trauma-related communication needs and gender-specific considerations, particularly in perinatal and mental health contexts [8,29]. Studies involving Sub-Saharan African migrants report linguistic distance and experiences of discrimination in relation to healthcare access and communication [11].

Evidence focusing on migrant women, particularly in maternity care, documents higher reported rates of communication difficulties compared with non-migrant populations [7,30]. Greek studies examining migrants with limited Greek proficiency report associations with longer waiting times, reduced comprehension of medical information, and lower satisfaction with care [10,12]. Group-specific findings and reported communication needs are summarized in Table 4.

**Table 4:** Differences in Communication Needs and Outcomes Across Migrant and Refugee Groups.

Population Group	Communication Challenges	Clinical Impacts	Specific Needs	Key Sources
Afghan refugees	Limited language proximity; cultural/gender norms	Lower comprehension; reduced consent	Intercultural mediation	[29]
Syrian refugees	Trauma-related communication needs; low literacy	Delayed histories; mistrust	Trauma-sensitive interpretation	[8]
Sub-Saharan African migrants	Low linguistic proximity; discrimination	Avoidance of care	Community-based mediators	[11]
Migrant women (perinatal)	High communication complexity	communication problems (35–55%)	Continuous interpreter support	[7,30]
Economic migrants with limited Greek	Partial comprehension, terminology gaps	preventive care use	Clear-language materials	[10,12]
Unaccompanied minors	No family advocates; trauma	Incomplete histories	Youth-trained mediators	[14]

## 5. Discussion

The findings of this review provide compelling, convergent evidence that language barriers constitute a critical determinant of health inequities for migrants and refugees in Greece. When interpreted alongside the European comparative data and the quantitative indicators presented in Table 1, Table 2, Table 3 and Table 4, it becomes clear that the communicative infrastructure of the Greek healthcare system remains significantly underdeveloped relative to both regional and international standards. Although Greece has articulated policy commitments to migrant and refugee health, the absence of a structured, state-funded interpretation system continues to impede safe, effective, and equitable care.

A central theme emerging from the synthesis is the unequivocal clinical value of professional interpretation. Quantitative studies consistently demonstrate that trained interpreters enhance communication accuracy and patient comprehension, reducing clinically significant errors by more than half and lowering hospital readmissions by nearly 40% [13,28]. As detailed in Table 2, these improvements are also associated with shorter inpatient length of stay and cost reductions of USD 2000–6000 per patient. These findings underscore that professional interpretation is not a peripheral convenience but a measurable determinant of patient safety, clinical quality, and system efficiency. By contrast, continued reliance on ad hoc interpreters—whether family members, bilingual staff, or untrained volunteers—introduces significant risks, including miscommunication, confidentiality breaches, and preventable adverse events [17,22]. The cumulative evidence suggests that reliance on ad hoc language support is associated with documented risks, while more structured approaches may offer advantages under specific organizational and resource conditions.

The cross-national comparisons presented in Table 3 further demonstrate that Greece’s challenges are neither inevitable nor insurmountable. European countries such as Italy, Spain, Germany, and Sweden have adopted diverse but well-structured models of interpreter provision—from Italy’s regional intercultural mediation programs to Sweden’s legal guarantee of free interpretation and Germany’s extensive tele-interpretation use. These models have produced measurable gains in patient satisfaction, adherence, and clinical outcomes. The contrast with Greek practices is particularly stark in perinatal care: while Swedish maternity services report interpreter use in 96% of encounters with migrant women [8], Greek facilities often rely on improvised or informal communication arrangements. This disparity highlights the feasibility and benefits of adopting systematic interpretation frameworks within the Greek public health system.

Another key finding of this review is that language barriers do not affect all migrant populations equally. As summarized in Table 4, Afghan and Syrian refugees face unique challenges due to linguistic distance, trauma histories, and gender norms, while Sub-Saharan African migrants often experience compounded communication and discrimination-related barriers [11,29]. Migrant women—particularly in perinatal settings—experience disproportionately high communication difficulties, with studies reporting 35–55% more communication problems compared to non-migrant women [7,30]. These findings underscore the need for intercultural mediators, who can address not only linguistic discordance but also cultural expectations, stigma, trauma-related needs, and gender-specific communication patterns. In Greek maternity wards, for example, persistent communication challenges have been reported by midwives and clinicians, highlighting the importance of specialized linguistic and cultural support during labor and childbirth [30].

Taken together, these results indicate that Greece’s continued reliance on informal and ad hoc interpreters represents a major barrier to equitable healthcare. Extensive international evidence suggests that such reliance undermines patient trust, satisfaction, and safety while producing systemic inefficiencies. Without institutional commitments to professional interpretation and intercultural mediation, language barriers will continue to perpetuate disparities in access, diagnostic accuracy, treatment adherence, and overall health outcomes.

In light of these findings, the review calls for a multi-level national strategy to address linguistic and cultural barriers in Greek healthcare. Key components include (a) integrating trained medical interpreters into clinical teams across primary, secondary, and emergency care; (b) expanding tele-interpretation and digital tools, particularly in rural and island regions; (c) establishing national guidelines or legal entitlements governing interpreter provision; and (d) embedding intercultural mediators to support refugee and migrant groups with complex cultural and psychosocial needs. Aligning Greek healthcare policy with successful European models would not only reduce inequities but also strengthen efficiency, safety, and patient trust.

Ultimately, the evidence assembled in this review underscores that linguistic support is not an auxiliary service but a fundamental prerequisite for delivering high-quality, culturally responsive healthcare in diverse societies. The quantitative and comparative findings presented across Table 1, Table 2, Table 3 and Table 4 highlight that systematic investment in professional interpretation and intercultural mediation is both ethically imperative and operationally advantageous. Strengthening Greece’s linguistic support infrastructure is therefore essential for advancing health equity and improving outcomes for the country’s growing migrant and refugee populations.

While much of the reviewed literature reports favorable associations between professional interpretation and healthcare outcomes, some studies also document mixed or context-dependent findings. Reported challenges include variability in interpreter availability, inconsistent integration into clinical workflows, and limited evidence on long-term cost-effectiveness across different health system models. In addition, the effectiveness of interpreter services may be moderated by factors such as legal status, institutional trust, provider attitudes, and patients’ health literacy. Acknowledging these constraints is essential to avoid overly deterministic interpretations and to situate interpretation services within broader structural and organizational conditions.

This review aims to provide both national specificity and international comparability. However, several limitations warrant acknowledgment. First, interpretation practices and documentation vary widely across Europe, limiting direct cross-country statistical comparison. Second, many Greek studies rely on self-reported data, which may introduce recall bias. Finally, grey literature and NGO reports—although essential for understanding the Greek context—may vary in methodological rigor.

Despite these limitations, the triangulation of quantitative clinical studies, qualitative research, and comparative policy analyses strengthens the reliability of the findings and offers a robust foundation for evaluating Greece’s current approach to linguistic and cultural mediation in healthcare.

The policy recommendations advanced in this review are grounded in the empirical patterns identified across the literature but should be interpreted in light of fiscal constraints, workforce capacity, and institutional readiness within the Greek healthcare system.

## 6. Practical Implications and Recommendations

Addressing language barriers in healthcare requires far more than isolated technical fixes; it demands a structural transformation of the Greek health system to ensure that communication needs are systematically identified and met. The findings of this review, together with comparative evidence from other European countries, indicate that Greece must adopt a comprehensive approach integrating professional interpretation, intercultural mediation, digital infrastructure, and community engagement. Such reforms align with international best practices and can generate measurable improvements in patient safety, efficiency, and equity.

In the short term, priority should be given to measures that ensure immediate visibility and response to linguistic needs within healthcare settings. A first essential step is the systematic identification and documentation of limited-language-proficiency (LLP) patients at the point of entry into the health system. Recording preferred language, interpreter needs, and cultural considerations within electronic health records enables clinicians to anticipate communication requirements and allows institutions to monitor the availability and quality of linguistic support [31,32]. Without such standardization, language barriers remain largely invisible, and communication failures continue to go unaddressed.

Concurrently, ensuring access to qualified linguistic support represents the most critical short- to medium-term intervention. Extensive international and Greek evidence demonstrates that trained medical interpreters and intercultural mediators substantially reduce miscommunication and improve clinical outcomes compared with ad hoc or family-based interpretation [5,17]. Countries such as Italy and Sweden have shown that institutionalized interpreter systems—whether delivered on-site or remotely—are associated with higher patient satisfaction, improved adherence, and enhanced safety. To align with these models, Greek healthcare facilities should establish both on-site interpreter rosters and remote (telephone or video) interpretation services. Remote interpretation, successfully implemented in Germany, offers a scalable and cost-effective solution for Greece’s island and rural regions and is compatible with ongoing digital-health initiatives. Investment in trained intercultural mediators is particularly important in high-demand settings such as maternity care, pediatrics, mental health, and emergency departments, where communication failures carry the greatest clinical risk [14,18].

In the medium term, strengthening workforce capacity is essential for the sustainable use of linguistic services. Linguistic accessibility depends not only on interpreter availability but also on healthcare professionals’ ability to collaborate effectively with interpreters and navigate cross-cultural encounters. Integrating cultural competence training and “working with interpreters” modules into undergraduate education and continuing professional development can enhance communication quality and reduce clinical risk [15]. In addition, assessing the language proficiency of bilingual staff before assigning them interpretation roles is necessary to prevent errors that may compromise patient safety [22].

Infrastructure and workflow integration constitute a complementary medium-term priority. The effective implementation of interpretation and mediation services requires access to private consultation spaces, inpatient telephones, and secure video-interpretation platforms that support confidentiality and patient comfort [33]. Dedicated interpreter access stations or mobile interpretation units in emergency and primary care settings can normalize interpreter use and facilitate rapid access during high-volume clinical encounters.

Digital solutions further extend the reach of linguistic support and should be expanded progressively. Telemedicine platforms, multilingual patient portals, and mobile health applications can support remote interpretation, appointment scheduling, medication adherence, and health education in patients’ preferred languages, particularly for migrants living in underserved, rural, or island areas [31]. Evidence suggests that such tools, when integrated with professional interpretation services, reduce missed appointments and improve chronic disease management.

Over the longer term, sustainable reform depends on community engagement and system-level accountability. Meaningful collaboration with migrant communities—including the co-design of health information materials, recruitment of mediators from relevant linguistic and cultural backgrounds, and clear communication of the right to interpretation—can enhance trust, reduce misinformation, and improve service utilization [10,12]. Partnerships with community organizations, as demonstrated in Spain and Italy, can further strengthen public health outreach and culturally grounded communication strategies.

Finally, Greece must establish mechanisms for continuous monitoring, evaluation, and accountability. Systematic tracking of indicators such as interpreter utilization rates, waiting times, readmissions, and patient satisfaction—disaggregated by language group—will support quality improvement and help identify persistent inequities requiring targeted intervention [13]. Such monitoring is consistent with international practice and is essential for demonstrating the clinical and economic value of professional interpretation.

Taken together, these measures constitute a phased, evidence-based strategy for strengthening linguistic and cultural accessibility in Greek healthcare. While implementation requires sustained investment, political commitment, and organizational change, the anticipated benefits are substantial: improved patient safety, enhanced satisfaction, reduced clinical errors, and more efficient use of healthcare resources. As Greece continues to serve diverse migrant and refugee populations, the establishment of a structured system of professional interpretation and intercultural mediation is not merely desirable but indispensable for delivering equitable, high-quality care.

## 7. Conclusions

This review demonstrates that language barriers represent a persistent and measurable source of inequity within the Greek healthcare system, disproportionately affecting migrant and refugee populations who already experience multiple vulnerabilities. Across diverse clinical settings, the evidence synthesized here consistently shows that limited proficiency in Greek undermines comprehension, reduces engagement in care, increases the risk of clinical errors, and contributes to inefficiencies within health services. Studies reviewed also indicate that the use of professional interpretation services—whether delivered in person or remotely—is associated with improved communication accuracy, shorter hospital stays, lower readmission rates, and higher patient satisfaction. Taken together, these findings confirm that structured linguistic support is a fundamental component of safe, effective, and patient-centered healthcare rather than an ancillary service.

The comparative perspective further situates the Greek case within a broader European context, illustrating that existing challenges largely reflect the absence of institutionalized language-support mechanisms rather than unavoidable systemic constraints. Evidence from Italy, Spain, Germany, and Sweden shows that coordinated interpreter provision and intercultural mediation are associated with more consistent access to care and improved utilization among migrant populations. These examples underscore the feasibility of structured approaches and provide relevant points of reference for policy development in Greece, without implying direct numerical transferability of outcomes.

The review also highlights that linguistic and cultural barriers are not experienced uniformly across migrant and refugee groups. Differences related to linguistic distance, gender, trauma exposure, and care context shape communication needs and clinical experiences. This synthesis reinforces the conclusion that linguistic accessibility must extend beyond literal translation to include intercultural mediation capable of supporting trust, cultural understanding, and context-sensitive communication.

Overall, the evidence assembled in this review supports the conclusion that the development of a coordinated interpretation and intercultural mediation framework constitutes both an ethical obligation and a practical requirement for advancing equity in Greek healthcare. Strengthening structured language support, in line with approaches documented in other European health systems, has the potential to improve safety, quality, and fairness in service delivery. Ensuring linguistically and culturally accessible care is therefore integral to the capacity of the Greek health system to respond effectively to the needs of migrant and refugee populations.

## Figures and Tables

**Table 1:** Comparative Outcomes by Interpreter Type.

Interpreter Type	Accuracy of Communication	Patient Satisfaction	Clinical Outcomes (LOS, Readmission)	Risks Identified
Professional Interpreters	High accuracy; fewer clinically significant errors [13,17]	Higher satisfaction for both patients and clinicians [25]	Reduced LOS; lower readmission; reduced costs [2,13]	Minimal; adherence to ethical standards
Intercultural Mediators	High accuracy plus cultural clarification [15,26]	High satisfaction, increased trust and engagement	Improved outcomes especially in mental health and perinatal care [18]	Requires sustained funding and training
Ad Hoc Interpreters	Low accuracy; frequent omissions/substitutions [17]	Mixed; often lower satisfaction [13]	Longer LOS; higher risk of errors and adverse outcomes [22,27]	Ethical concerns; confidentiality risks

## Data Availability

No new data were created or analyzed in this study. Data sharing is not applicable to this article.

## Abbreviations

The following abbreviations are used in this manuscript:

CCC	Culturally competent care
IOM	International Organization for Migration
LEP	Limited English proficiency
LLP	Limited Language Proficiency
NCIHC	National Council on Interpreting in Health Care
OCR	Office for Civil Rights (U.S. Department of Health and Human Services)
WHO	World Health Organization
